# Right diaphragmatic iatrogenic hernia after laparoscopic fenestration of a liver cyst: report of a case and review of the literature

**DOI:** 10.1186/1749-7922-8-2

**Published:** 2013-01-03

**Authors:** Mehdi Soufi, Hélène Meillat, Yves-Patrice Le Treut

**Affiliations:** 1 Division of digestive Surgery and transplantation, Hopital de la Conception, Marseille, France; 2 Faculty of medicine, University Mohammed First, Oujda, Morocco; 3 Division of digestive Surgery, Oujda CHU, University Mohammed first, Faculty of medicine Bp 4847 Oujda University, 60049, Oujda, Morocco

**Keywords:** Diaphragm injury, Iatrogenic right diaphragmatic hernia, Laparoscopic Fenestration of liver cyst, Central tendon of the diaphragm

## Abstract

Iatrogenic right diaphragmatic hernia is very rare. We report the first case of a patient who had a diaphragmatic hernia after laparoscopic fenestration of liver cyst. A herniorrhaphy of the diaphragmatic defect was carried out after reducing the herniated organ. The postoperative course was uneventful. Diaphragmatic hernias are not as common as the traumatic type. Surgeons can easily miss diaphragmatic injuries during the operation especially after laparoscopy. Late diagnosis of iatrogenic diaphragmatic hernias is frequent. Ct scan is helpful for diagnosis. Surgery is the treatment of diaphragmatic hernia at the time of diagnosis, even with asymptomatic patients. The incidence of iatrogenic diaphragmatic hernia after surgery may be reduced if the surgeon checks for the integrity of the diaphragm before the end of the operation. A review of the literature is also performed regarding this rare complication.

## Introduction

Liver cysts are benign congenital malformations resulting from isolated aberrant biliary ducts [1]. Laparoscopic fenestration is the treatment of choice for symptomatic simple liver cysts. The indication for surgery should be limited to symptomatic, which involves 5% to 10% of all liver cysts [2]. Acquired diaphragmatic hernias are generally the result of blunt or penetrating thoraco-abdominal trauma or iatrogenic injury [3]. Postoperative iatrogenic diaphragmatic hernia right is very rare. We describe a iatrogenic right diaphragmatic hernia after laparoscopic fenestration of right liver cyst.

## Case report

**Figure 1:**
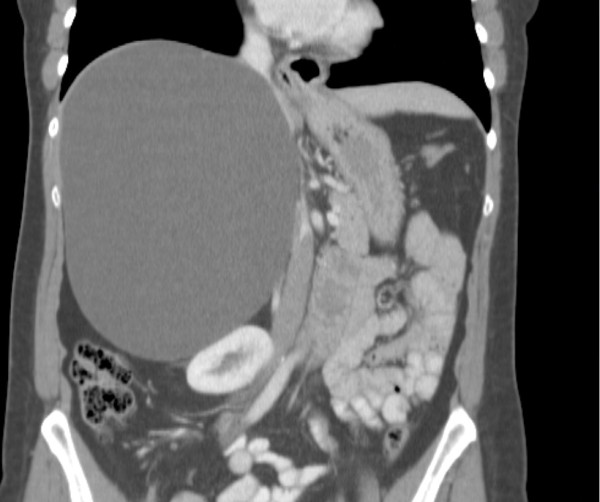
CT scan showing the 20 x 14 cm simple liver cyst.

**Figure 2:**
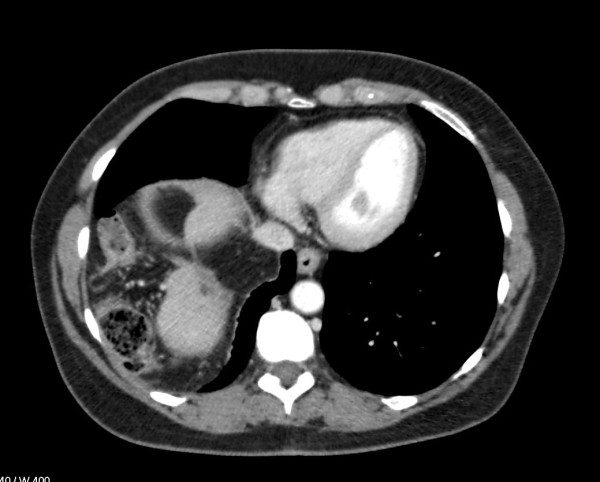
CT scan Transversal computed tomography (CT) showing the loop of colon in the right-sided diaphragmatic hernia.

**Figure 3:**
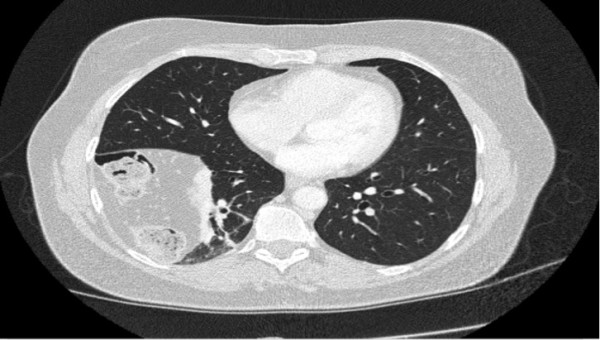
CT scan Transversal computed tomography (CT) showing the loop of colon in the right-sided diaphragmatic hernia.

A 61-year-old female with a past medical history of laparoscopic fenestration, one year ago, of a huge right liver benign cyst (Figure 1) presented to our department with right upper abdominal and thoracic pain without vomiting. Chest x-ray showed an elevated right hemidiaphragm. Abdominal examination was normal. Computed tomography CT- scan showed a right posterior diaphragmatic hernia and passive atelectasis due to an ascent of the colon with corresponding mesos and Omentum in the chest cavity (Figures 2 and 3). Laboratory tests showed no abnormality. After coeliotomy through right subcostal incision and reduction of the herniated organs, a defect 10 cm in diameter was found at the central tendon of the right diaphragm. Direct herniorrhaphy of the diaphragmatic defect was easily carried out. The patient had an uneventful postoperative recovery and the thoracic drain was removed on the second postoperative day. The patient was discharged on the seventh postoperative day.

## Discussion

Surgery is the mainstay of therapy in benign liver cyst. Lin and al [4] first described the technique of fenestration or deroofing of the cysts in 1968 and laparoscopic fenestration was first reported in 1991. Many complications have been reported such as bile leakage, ascites and pleural effusion [5]. In our knowledge, a right diaphragmatic hernia after laparoscopic fenestration of a liver benign cyst had never been reported in the literature review. It’s the originality of our case.

The diaphragmatic hernia is a herniation of abdominal structures within the thoracic cavity. It can be either congenital or acquired. Diaphragmatic acquired defects are most commonly traumatic in origin, followed by iatrogenic lesions and spontaneous defects [3]. These are usually on the left side, attributed to the cushioning effect of the liver protecting the right hemidiaphragm [3]. Right-sided traumatic diaphragmatic hernias are more often related to penetrating injuries, but may also occur as a complication of surgery. Iatrogenic right diaphragmatic hernias have been reported after laparoscopic cholecystectomy [6], laparoscopic hepatectomy [7], splenectomy [8], laparoscopic gastric banding [9] splenopancreatectomy [10], gastrectomy [11] and after living donor liver transplant [12,13]. Mostly, this complication has been known to develop after esophagectomy and nephrectomy [14-17] (Table 1).

**Table 1:** The characteristics of the reported cases of iatrogenic diaphragmatic hernia

**Case**	**Age**	**Gender**	**Time to diagnosis**	**Initial surgical procedure**	**Localisation of defect**	**Surgical procedure**	**Year**
1[6]	53	Women	6 weeks	Laparoscopic cholecystectomy	Right	Thoracotomy	1999
2[7]	31	Women	9 months	Laparoscopic hepatectomy	Left	Thoracotomy	2003
3[8]	35	Women	24 months	Laparoscopic gastric banding	Left	Laparotomy approach	2008
4[9]	60	Man	6 weeks	Splenectomy for Hydatid cyst	Left	Thoracotomy	2010
5 [10]	51	Man	4 years	Splenopancreatectomy	Left	Thoracotomy	2006
6[11]	81	Women	8 months	Laparoscopy assisted total Gastrectomy total	Left	Laparoscopy	2012
7[12]	44	Man	28 months	Living donor liver transplant	Right	Laparotomy approach	2010
8[13]	54	Man	3 years	Right donor and Hepatectomy	Right	Thoracotomy	2006
9[14]	50	Man	6 months	Nephrectomy	Left	Thoracotomy	1995
10[15]	74	Man	5 years	Nephrectomy	Right	Thoracotomy	1996
11[16]	69	Man	3 years	Nissens procedure	Left	Thoracotomy	1996
11[18]	39	Women	35 years	Transthoracic oesophagogastrectomy	Left	Laparotomy	1988
12[19]	47	Women	1 day	Nephrectomy	Left	Thoracotomy	2008
13[24]	60	Man	4 months p	Lung resection	Left	Thoracotomy	2010
14[25]	19	Women	2 years	Lower lobectomy	Left	Laparoscopy	2000
Current study	61	Women	1 year	Laparoscopic fenestration right liver benign cyst	Right	Laparotomy	2012

A late presentation of a iatrogenic hernia diaphragm was reported in 5%–62% of cases in different series, with the longest reported delay of 35 years [18].

Grasping instrument and electrocautery and dissection near of the diaphragm may cause diaphragmatic injuries after surgery. The energy generated by these instruments especially the ultrasonic activated scissor can provoke a weak point in the diaphragm [11].

That’s why surgeons must be careful handling the instruments, thermofusion and ultrasonic dissector during laparoscopy [6,19]. A small diathermy injury may not be observed during surgery; any such defect in the diaphragm is likely to increase in size as a result of the gradient of pressure between the abdominal and pleural cavities. This is what probably happened in our patient who had a 10 cm defect.

Patients with large diaphragmatic defects can have critical problems shortly after surgery due to cardiorespiratory disturbances. Unexplained pain in post operative is not specific but should suspect this complication. Other patients may be asymptomatic or have vague symptoms, which may delay the diagnosis. Our patient presented pain one year after the first surgery. The diagnosis of a cyst recurrence was suspected firstly but not the diagnosis of a diaphragmatic hernia.

The clinical features are usually chronic symptoms such as upper abdominal and lower chest pain, nausea, dyspnea, and reflux after meals, which may develop into an acute presentation with severe epigastric pain, vomiting, and intestinal obstruction [11,19].

The radiological diagnosis is often complex and includes several imaging modalities [18]. Chest radiograph is a good screening examination, but only 50% of patients show an abnormality [18,19]. CT scan is the best imaging modality to diagnose diaphragmatic hernias. Its sensitivity is high but specificity is only 50% for the right side [20,21]. Surgery is the treatment of diaphragmatic hernia at the time of diagnosis, even in asymptomatic patients.

Some authors think that the thoracotomy is the elective surgical approach that can correct anatomical restoration of the chest and abdominal cavity especially when it is the approach during the initial surgical procedure [22-24]. Though patients who had a thoracotomy approach had the longest length of stay with a higher need for postoperative mechanical ventilation than those undergoing an abdominal approach after diaphragmatic hernia repair. Paul et al. found that the thoracotomy approach is an independent predictor of the development of a pulmonary embolism [25]. We think that laparotomy through a right subcostal incision is a more efficient approach into the abdominal cavity.

Treatment by laparoscopy is feasible with a shorter length of stay. This approach is especially used in left diaphragmatic hernia repair [11,26].

Because of liver bulk, right side hernia is not amenable to laparoscopic repair, with a high level of conversion. However some authors described this approach with success [27]. In our patient, the hernia was in the right side of hepatic vein, this was the reason we preferred a laparotomy approach.

Herniated contents are reduced, the muscular defect is treated and an endothoracic drain is placed [28]. In some cases a bowel resection might be needed in case of ischemia. The mortality following emergency surgery for these cases rises between 20% and 80%. [14,15].

The majority of these defects can be repaired safely with non-absorbable sutures without the need for a prosthetic mesh [21,28]. With an increase in the number of laparoscopic surgery performed, it is likely that this complication will increase. It is therefore important that surgeons be aware of this potentially serious complication by looking to the diaphragm in the end of each surgical procedure [29]

## Conclusion

Iatrogenic herniation of abdominal contents after laparoscopic fenestration of liver cyst is a rare complication. Iatrogenic diaphragmatic injury can be missed during surgery. Surgeon must take precaution to avoid it by precise dissection when using the instruments during surgery. The incidence of iatrogenic diaphragmatic hernia after surgery may be reduced if a final look of diaphragm is systematically realized at the end of each laparoscopic operation.

## Consent

Written informed consent was obtained from the patient for publication of this case report and accompanying images.

## Abbreviation

CT: Computed tomography.

## Competing interest

All Authors does not have any financial relationship with any organization. No benefits in any form have been received or will be received from a commercial party related directly or indirectly to the subject of this article. All authors have the full control of all primary data and that they agree to allow the journal to review their data if requested. All authors contributed to the realization of this manuscript. The authors declare that they have no competing interests.

## Authors’ contributions

All of the authors were involved in the preparation of this manuscript.MS write the manuscript coordinated the team, and helped in literature research. HM was an assistant surgeon and made substantial contributions to conception and design. YPL performed the operation and edited the final version of the manuscript. All authors read and approved the final manuscript.
